# Older patients with proximal femur fractures and SARS-CoV-2 infection – An observational study

**DOI:** 10.1051/sicotj/2021001

**Published:** 2021-02-17

**Authors:** Abdus S Burahee, Veronica E Barry, Robert P Sutcliffe, Sabreena Mahroof

**Affiliations:** 1 Department of Orthopaedic Surgery, Royal Wolverhampton NHS Trust WV8 1DN Wolverhampton UK; 2 Liver Unit, Queen Elizabeth Hospital Birmingham B15 2WB UK

**Keywords:** Proximal femur fracture, Elderly Care, COVID-19, SARS-CoV-2, Vitamin D

## Abstract

*Background***:** Older patients are at increased risk of severe COVID-19 infection and associated mortality. There are limited data evaluating the outcome of older patients with hip fractures treated during the COVID-19 pandemic, and it has been suggested that these patients should be treated non-operatively due to high mortality risk. The aim of this study was to report the outcomes of COVID-19 infected hip fracture patients treated at a single centre. *Methods*: This was a retrospective cohort study. Data were collected from February 2020 (after the first confirmed COVID-19 infected patient was reported in the Midlands region of the UK). All patients admitted to the hospital with femoral neck fractures were included. Patient demographics, comorbidity, COVID-19 status, and short-term clinical outcomes were obtained by review of electronic medical records. The outcomes of COVID-19 infected patients were compared with non-COVID-19 patients treated during the study period. *Results*: Twenty-nine patients were included (mean age of 80 years), of whom 14 (48%) were tested positive for COVID-19 infection in the postoperative period. Overall, 26 patients (90%) underwent surgical treatment. COVID-19 infected patients had significantly higher Charlson comorbidity scores compared to the control group (5 vs. 4; *p* = 0.047). Only 5 COVID-19 infected patients (36%) required supplemental oxygen therapy in the postoperative period, and no patients required respiratory or other organ support. The 30-day mortality rate in COVID-19 patients was 14% compared to 0% in the negative controls (*p* = 0.22). *Interpretation*: COVID-19 infection did not increase the mortality rate of older patients undergoing surgery for hip fractures during the pandemic. The authors recommend careful assessment of patient fitness and prompt surgical treatment. In addition, it was noted that nearly all admissions were either given large boluses of Vitamin D or were on maintenance supplementation, which may have affected the severity of the response to COVID-19 infections.

## Introduction

In December 2019, the World was introduced to a novel coronavirus disease (COVID-19) that originated in China and subsequently developed into a pandemic. COVID-19 has been attributed to the Severe Acute Respiratory Syndrome Corona Virus 2 (SARS-CoV-2) and is characterized by viral pneumonia, associated with fever, cough, sore throat, myalgia, and fatigue [1]. Although the majority of cases of COVID-19 are mild and self-limiting, European data shows 32% of patients require hospitalisation, and 2.4% require respiratory support and/or ventilation. The crude fatality rate was 1.5% in diagnosed cases and 11% among hospitalisation cases. The chances of hospitalisation, severe illness, and death increases over the age of 65 and with defined comorbidities (hypertension, diabetes, cardiovascular disease, chronic respiratory diseases, compromised immune status, cancer, and obesity) [2]. Several risk factors have been associated which an increased risk of mortality with COVID-19, including age and comorbidity [2].

Hip fractures are common and typically occur in older patients who often have significant comorbidity [3]. Hip fractures in older patients carry a significant mortality rate, and it is well established that patients should undergo urgent surgery to facilitate early mobilisation and reduce morbidity [3]. Despite concerns that older patients with hip fractures may be particularly susceptible to developing severe COVID-19 infection in the perioperative period, there is currently little evidence to support this. At present, there is limited data available on the outcome of fracture patients with COVID-19 infection. A recent small study from Wuhan, China reported a mortality of 40% for patients with COVID-19 infection and concomitant fractures, and the authors recommended non-operative management of older patients with hip fractures [4]. The aim of this study was to report the outcomes of a consecutive series of COVID-19 infected patients who were hospitalized with hip fractures.

## Patients and methods

This was a retrospective cohort study undertaken at The Royal Wolverhampton NHS Trust, UK, which was described as the epicentre of the pandemic within the Midlands region of the country [5]. The study was registered on the departmental audit database. Consecutive patients who were admitted with a diagnosis of femoral neck fracture between 1st March and 7th April 2020 were identified from the Orthopaedic Department prospective database. Patients were grouped according to the presence or absence of COVID-19 infection during their hospital stay and their outcomes were compared. The following data was collected for each patient by retrospective review of medical records: patient age and demographics, comorbidity, ASA grade, operation performed, postoperative vital signs, and laboratory results, complications and length of hospital stay. Final outcomes were assessed at a minimum of 30 days after a positive test or surgery. COVID-19 infection was confirmed by quantitative RT-PCR analysis of upper respiratory tract swabs. Lymphopenia was defined as a sustained differential count of less than 1 × 10^9^/L for three consecutive days. The Nottingham Hip Fracture Score, which estimates 30-day mortality postoperatively using a summative score of seven preoperative variables [6], was calculated for each patient. This gives a score ranging from less than 1% (score = 0) to 45% (score = 10).

### Statistical analysis

Continuous variables were represented as the median and interquartile range (IQR). These were compared using an independent group test or Mann-Whitney test as applicable. Categorical data were expressed as frequencies and percentages and compared by Pearson’s Chi-Square test or Fisher’s exact test as applicable. A two-sided *p*-value of less than 0.05 was considered statistically significant. All analyses were performed with SPSS software (Version 26).

## Results

Twenty-nine consecutive patients were admitted with femoral neck fractures during the study period, of whom 19 (66%) were female gender. The mean age of patients was 80 years (range 56–96). The median ASA grade was 3 (range 1–4) and the median Charlson comorbidity score was 4 (range 1–9). The clinical outcomes of all patients were monitored for 2 months post-injury. No patients were lost to follow-up during this period. Of the entire cohort, 26 patients (90%) underwent surgery (dynamic hip screw 11, hemiarthroplasty 10, femoral nail 3, total hip replacement 1, screw fixation 1). Three patients were treated conservatively. Two had stable fracture configurations; one had been mobilising with what was deemed to be an old fracture and the other had an impacted fracture with no pain on examination and weight-bearing. One patient was deemed to be unfit for an anaesthetic. Surgery was performed under general anaesthesia in 10 patients (38%) and regional anaesthesia in 16 (62%). During the study period, 14 patients (48%) had a spike in temperature during their inpatient stay and all tested positive for COVID-19 infection after a median of 10 days after admission (range 4–35), the majority of whom had undergone surgery (11 patients; 79%). Interestingly, the COVID-19 infected patients had a significantly higher Charlson comorbidity score compared to negative controls (Table 1). Of the COVID-19 infected patients, 5 (36%) required supplemental oxygen therapy but none required respiratory or other organ support. Seven COVID-19 infected patients (50%) had lymphopenia. There were two deaths in COVID-19 infected patients attributed to the disease after either surgery (*N* = 1) or conservative management (*N* = 1). During the same period, 15 patients admitted with femoral neck fractures did not present with nor developed symptoms compatible with COVID-19 infection. They acted as the control group in this study. These patients were not tested and were deemed to be COVID-negative on the basis of (i) no respiratory symptoms and no supplemental oxygen requirement, (ii) chest radiograph performed on admission, and (iii) baseline blood tests including inflammatory markers done as part of our local femur fracture admission protocol. During the early phase of the pandemic, the National guidelines did not require patients to be routinely screened using COVID swabs for PCR prior to admission. One patient in this group developed a COVID-19 infection after hospital discharge and died one month later. COVID-positive and COVID-negative patients had similar ages, comorbidity, and median Nottingham Hip Fracture Scores. There was no significant difference in outcomes between the two groups. The 30-day mortality rate in COVID-19 patients was 14% (*N* = 2) compared to 0% in the negative controls (*p* = 0.22).

**Table 1:** Comparison between COVID-positive and negative femoral neck fracture patients.

	COVID-19 positive (*N* = 14)	COVID-19 negative (*N* = 15)	*P*-value
*Demographics*			
Median age (years)	82 (67–96)	79 (56–93)	0.47
Female gender	7 (50%)	12 (80%)	0.13
ASA grade	3 (2–4)	3 (1–4)	0.98
Charlson Comorbidity score	5 (3–8)	4 (1–9)	0.047
*Fracture management*			
Nottingham Hip Fracture score	5.5 (4–7)	4.4 (1–7)	0.09
Surgery	11 (79%)	15 (100%)	0.10
Conservative treatment	3 (21%)	0 (0%)	0.10
*Outcomes*			
Median length of hospital stay (range)	19 (7–40)	11 (6–18)	0.11
Hospital mortality	2 (14%)	1 (6%)	0.22
30-day mortality	2 (14%)	0 (0%)	0.22

All patients arriving into the department had a serum Vitamin D level routinely performed as part of our femoral fracture protocol. Based on the fact that patients who sustain a fracture in a low energy fall are usually deficient in Vitamin D, our treatment policy is to treat these patients on admission. We would routinely load patients prophylactically with 100,000 units over four days in the immediate postoperative period and a further 200,000 units over four weeks if serum Vitamin D levels demonstrate an insufficiency (<30 nmol/L). Twenty-six patients had Vitamin D levels performed and were receiving supplementation during their stay due to insufficiency. Three patients were not flagged up during admission due to clerical errors and missed Vitamin D level testing and supplementation.

The demographics and outcomes of each group are summarised in Table 1.

## Discussion

This small early study has demonstrated that the presence of COVID-19 infection in the postoperative period did not significantly increase the mortality rate of patients admitted with hip fractures during the COVID-19 pandemic compared to an internal control group or data from the UK National Hip Fracture database [7]. The vast majority of patients in this study were older with significant medical comorbidity, typical of patients who sustain hip fractures, and would be considered at high risk of mortality from COVID-19 [8]. Two COVID-19 infected patients in this study died, although the cause of death in both patients was unknown and not definitively due to COVID-19. It is not clear whether patients were COVID-19 positive at the time of hospital admission since testing was only performed in the postoperative period in patients who developed potential COVID-19 related symptoms. All COVID-19 positive patients were diagnosed at a minimum of four days after hospital admission. The majority of patients (64%) were diagnosed with COVID-19 infection later than one week after admission, suggesting that most patients probably acquired COVID-19 during their hospitalisation, although it is possible that some patients had asymptomatic COVID-19 at the time of hip fracture. Interestingly, the majority of patients who were diagnosed with COVID-19 infection had a mild, self-limiting disease since only a third of patients required supplemental oxygen therapy and no patients required high dependency or intensive care admission.

It is not clear why this group of high-risk patients developed a milder form of COVID-19 infection, although there are several possible explanations. There have been several reports of high mortality associated with surgery in COVID-19 infected patients [9], including a study from Wuhan that reported a mortality of 40% in patients with fractures [4], and surgeons have been cautioned against undertaking high-risk elective surgery during the COVID-19 pandemic. However, a recent study of hip fracture patients who were COVID-19 infected in Italy demonstrated similar outcomes to this study with a 25% mortality [10].

The severity of COVID-19 infection may be influenced by the host immune response, and a cytokine storm is considered important in the pathophysiology of respiratory and/or multi-organ failure associated with severe cases [11–13]. Although immunosenescence is well recognized in the older population [14, 15], there is also evidence that circulating pro and anti-inflammatory cytokines, such as Interleukin-6 (IL-6) and Interferon-Gamma (IFN-g), are uniquely deranged in patients after hip fracture resulting in immunosuppression leading to increased risk of bacterial infections [14–16]. IL-6 (a pro-inflammatory cytokine and anti-inflammatory myokine) is usually raised after a fracture. In addition to this serum IFN-g (also a pro-inflammatory cytokine, involved in immunomodulation and inhibiting viral replication) is reduced in these circumstances. TRAIL receptors (Tumour Necrosis Factor Related Apoptosis-Inducing Ligand) involved in the inflammatory response and a protective factor in pulmonary diseases is reduced in this group particularly after surgery. It is therefore feasible that the pathophysiology of hip fractures may involve an anti-inflammatory cytokine response, which could potentially protect such patients against developing severe immune-mediated complications of COVID-19.

We would also like to suggest an alternative hypothesis to explain the relatively low mortality associated with COVID-19 in this patient cohort. There is emerging evidence that Vitamin D supplementation may improve the outcome of patients infected with COVID-19 [17], and a preponderance of Vitamin D deficiency among patients of BAME (Black, Asian, and Minority Ethnic) origin may explain an increased severity of COVID-19 disease in this subgroup [18]. The majority of patients who sustain hip fractures have underlying osteoporosis and Vitamin D deficiency [3], and routine Vitamin D supplementation is recommended for all patients admitted with a hip fracture [3]. Ninety percent of patients (*N* = 26) in this study were either given or were receiving Vitamin D supplementation in the perioperative period, and this may have been an important factor in protecting them against severe infection. The two patients who died in the COVID 19 positive group did not receive Vitamin D supplementation while the third patient was in the control group and made a full recovery. Vitamin D plays an important role in immunomodulation of both the active and passive immune systems via Toll-Like receptors (TLR) which are expressed on Antigen-presenting cells (APC), T cells and B cells. However, the mechanism by which Vitamin D is thought to improve the outcome of COVID-19 infection is currently unknown, but recent publications have recommended Vitamin D supplementation for all infected patients [17]. To our knowledge, this is the first paper that has commented on Vitamin D supplementation dosage in COVID-19 infected patients with hip fractures and may demonstrate a simple process applicable to the management of COVID-19 infected patients without a hip fracture.

In conclusion, this study suggests that the mortality rate after hip fracture surgery during the COVID pandemic is not significantly increased, and that hip fractured patients may be protected from developing severe COVID infection. Due to the small sample size and retrospective nature of this study, it is not possible to draw firm conclusions about any potential reasons for the relatively low mortality rate that was observed in this study and this should be interpreted with caution.

Further work is warranted to investigate the unusual immune response in hip fractured patients and its effect on COVID-19 infections. Further studies are required to evaluate the potential effect of Vitamin D supplementation in patients infected with COVID-19, which may be facilitated by data already collected in the National Hip Fracture Database.

## Authors contribution

AB, VB, and SM shared the idea for and designed the study. AB and VB collected the data. All named authors had full access to all the data. AB drafted the manuscript. RPS did the analysis and all authors critically revised the manuscript for important intellectual content and gave approval for the final version to be published.

## Transparency declaration

The lead author affirms that this manuscript is an honest, accurate, and transparent account of the study being reported; that no important aspects of the study have been omitted; and that any discrepancies from the study as planned (and, if relevant, registered) have been explained.

## Conflict of interest

AB certifies that he or she has no financial conflict of interest (e.g., consultancies, stock ownership, equity interest, patent/licensing arrangements, etc.) in connection with this article.

VB certifies that he or she has no financial conflict of interest (e.g., consultancies, stock ownership, equity interest, patent/licensing arrangements, etc.) in connection with this article.

SM certifies that he or she has no financial conflict of interest (e.g., consultancies, stock ownership, equity interest, patent/licensing arrangements, etc.) in connection with this article.

RPS certifies that he or she has no financial conflict of interest (e.g., consultancies, stock ownership, equity interest, patent/licensing arrangements, etc.) in connection with this article.

## Ethical approval

Ethical approval was not required for the conduct of this study.

## Funding

The authors declare no source of funding.
